# Soft and broadband infrared metamaterial absorber based on gold nanorod/liquid crystal hybrid with tunable total absorption

**DOI:** 10.1038/srep16698

**Published:** 2015-11-18

**Authors:** Zhaoxian Su, Jianbo Yin, Xiaopeng Zhao

**Affiliations:** 1 Smart Materials Laboratory, Department of Applied Physics, Northwestern Polytechnical University, Xi’an, 710129, China

## Abstract

We design a soft infrared metamaterial absorber based on gold nanorods dispersed in liquid crystal (LC) placed on a gold film and theoretically investigate its total absorption character. Because the nanorods align with the LC molecule, the gold nanorods/LC hybrid exhibits different permittivity as a function of tilt angle of LC. At a certain tilt angle, the absorber shows an omnidirectional total absorption effect. By changing the tilt angle of LC by an external electric field, the total absorption character can be adjusted. The total absorption character also depends on the concentration, geometric dimension of nanorods, and defect of nanorod arrangement in LC. When the LC contains different size of gold nanorods, a broadband absorption can be easily realized. The characteristics including flexibility, omnidirectional, broadband and tunablility make the infrared metamaterial absorber possess potential use in smart metamaterial devices.

Electromagnetic metamaterials pave a new way to control the electromagnetic wave as people’s will^1^,^2^. Up to now, many kinds of metamaterials that can reflect, bend, or absorb light with no diffraction have been fabricated^3^,^4^,^5^. Metamaterial absorber is one of the most important metamaterials, owing to their unique property of total absorption to electromagnetic waves and promising applications in sensors, solar cells, and thermal emitters^6^,^7^,^8^,^9^. Since Landy *et al.* fabricated the first microwave metamaterial absorber^6^, many efforts have been made to achieve various metamaterial absorbers at different frequencies including microwave^10^, terahertz^11^, infrared^12^, and visible optical regions^13^. However, most of the metamaterial absorbers have been fabricated by arraying periodically resonant units on hard solid matter. Once they have been fabricated, the structures of these absorbers can’t be further changed or adjusted. As a result, the response frequency and intensity of the absorber are fixed, i.e. the absorbers are lack of flexibility and tunability.

To extend the application of metamaterial absorbers, the realization of tunability is very important. Some researchers have designed switchable metamaterial absorbers in microwave region by introducing diodes into the structure of absorbers^14^. However, coupling optical devices with diodes is almost impossible due to the difficulty in the sample preparation. Introducing graphene sheets into metamaterial absorbers is also one way to tuning the absorption^15^,^16^, because the Fermi level and conductivity of graphene sheets can be adjusted by electric or magnetic fields^17^,^18^,^19^. However, the adjusted frequency range is limited into far infrared and terahertz region. Furthermore, the fabrication of the graphene-based absorbers with desired resonance response is also difficult because it is lack of large scale and fine preparation methods. Compared to hard solid matter, soft matter not only has more flexibility but also can change its structure and behavior under external stimuli because a long-range self-organized or ordered structure of molecules or particles is easy to form and adjust in soft matter under external stimuli^20^. Among various soft matter, liquid crystal (LC) may be the most promising candidates due to its electrically or magnetically tunable optical property^21^,^22^,^23^,^24^,^25^. Some recent researches have proposed metamaterial absorbers loaded with LC to achieve the regulation of absorption intensity and bandwidth. For example, Shrekenhamer *et al.* have demonstrated an electronically tunable metamterial absorber by incorporating active LC into strategic locations within the metamaterial unit cell and realized the absorption modification of 30% at 2.62 THz, as well as the adjustment of resonant absorption over 4% in bandwidth^26^. Savo *et al.* have fabricated a terahertz spatial light modulator by coupling metamaterial absorber with isothiocyanate-based LC, which shows an overall modulation depth of 75%^27^. All these studies only have regarded LC as an additive or filler to the solid metamaterial absorbers, however, the total metamaterial absorbers are still solid.

In this paper, we propose a novel soft metamaterial absorber based on gold nanorods/LC hybrid placed on a gold thin film. Here, the LC of smectic c phase is used because it has a tilt angle and the tilt angle can be adjusted by an external electric field^28^,^29^. According to the previous reports^30^,^31^, the tilted array of nanorods is benefit for decreasing the thickness of polarization-dependent terahertz plasmons or other metamaterial devices. Thus, when the gold nanorods are dispersed into the LC of smectic c phase, the nanorods will align with the LC molecule and we can change the orientation of gold nanorods by adjusting the tilt angle of LC by external electric field^32^. As a result, the permittivity of the gold nanorods/LC hybrid will change with the tilt angle of LC and orientation of gold nanorods. At a certain tilt angle, the absorber shows a total absorption behavior due to impedance match. The total absorption is nearly omnidirectional but the position of absorption peak depends on the geometric dimension and volume fraction of gold nanorods in LC. When different length of nanorods are added in the LC, we can realize a broadband total absorption.

## Results and Discussion

### Theory analysis

**Figure 1:**
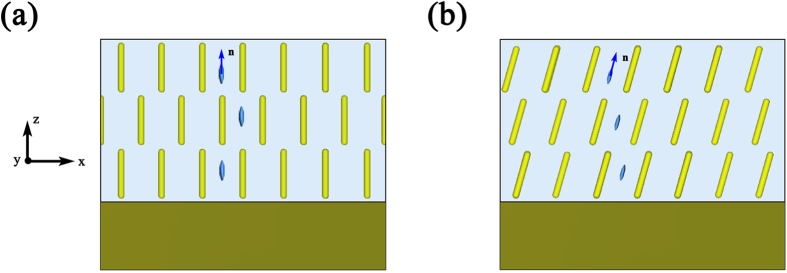
The schematic structure of soft gold nanorods/LC hybrid absorber at different tilt angle: (**a**) *φ* = 0. (**b**) *φ* ≠ 0.

Figure 1 shows the schematic structure of the soft gold nanorods/LC hybrid absorber. The absorber is composed of gold nanorods dispersed in LC of smectic c phase placed on a gold film. Here, the gold film acts as not only a reflection surface but also an electrode so that an external electric field can be applied to adjust the tilt angle of LC. According to the previous study about nanorods dispersed in LC, the nanorods will align with the direction of LC molecule^32^. As shown in Fig. 1(a), the nanorods align with z axis when the direction of LC molecule (unit vector **n**) is along z axis (i.e. *φ* = 0). When the direction of LC molecule shows a tilt angle 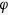 with z axis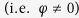, the orientation of nanorods will tilt with the same angle, as shown in Fig. 1(b). When 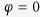, the gold nanorods/LC hybrid is an anisotropic medium due to the inward anisotropy of LC and alignment of nanorods and its permittivity can be described as 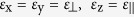. The subscript 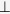 represents the direction perpendicular to the unit vector **n**, the subscript 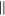 represents the direction parallel to the unit vector **n**. When 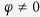, the permittivity of the hybrid in the Cartesian coordinate system (x, y, z) will be combined with rotation matrix *R*:


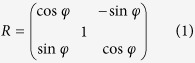


Thus, the permittivity can be written as:


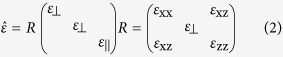


Such hybrid system can be regard as an asymmetric uniaxial medium. Assuming a harmonic time dependence exp(−i*ωt*), from Maxwell’s equations we have the expression of the normal component of the wave vector (*k*_z_) for Transverse Magnetic (TM) waves^31^:


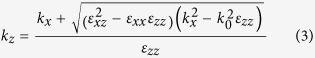


And its impedance can be derived as


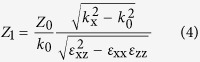


where 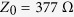 is the free-space impedance. Thus, the effective impedance (*Z*_*eff*_) of the gold nanorods/LC hybrid absorber can be given by the following Eq. (5):


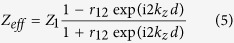


where 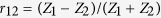, 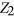 is the impedance of the gold film, *d* is the thickness of the gold nanorods/LC hybrid.

Based on the description above, it can be found that the effective impedance of the absorber has a dependence on the tilt angle 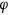 of LC. At different tilt angles, the permittivity of the hybrid will be different. Therefore, the effective impedance of the absorber will change as a function of tilt angle 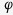. At a proper tilt angle, the effective impedance of the absorber will match with that of the free-space impedance and the absorber will show a total absorption effect. In particular, because the tilt angle 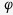 of LC can be simply adjusted by an external electric field, the absorption intensity of the absorber will be tunable by an external electric field.

### Simulation results

**Figure 2:**
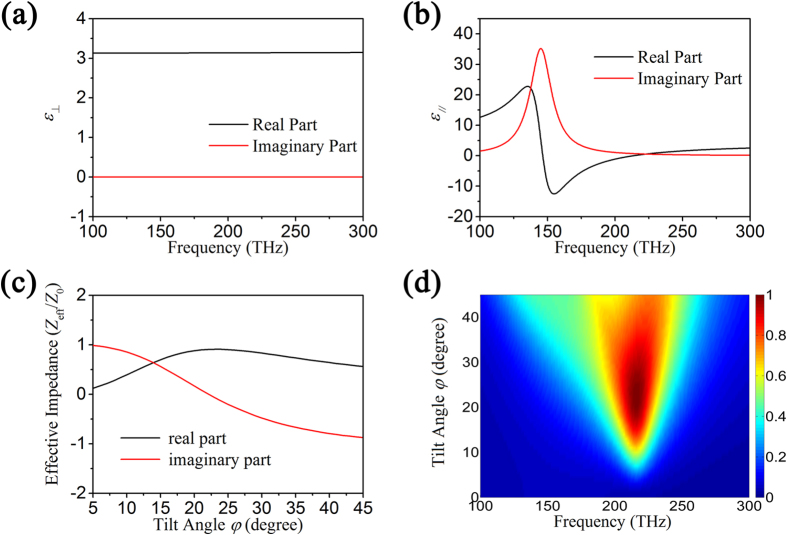
(**a**) The permittivity of the gold nanorods/LC hybrid perpendicular to the unit vector **n**; (**b**) The permittivity of the gold nanorods/LC hybrid parallel to the unit vector **n**; (**c**) The effective impedance of the gold nanorods/LC hybrid as a function of tilt angle at 215 Hz; (**d**) The absorption intensity of the gold nanorods/LC hybrid absorber at different LC tilt angles for different incident frequencies. (*p* = 0.021).

We first simulate the absorption spectrum of the gold nanorods/LC hybrid absorber through effective medium approximation. Here, the radius and length of the gold nanorods that we employ in the absorber are *r* = 4.95 nm and *l* = 77 nm, respectively. We assume the relative permittivity components of LC are 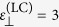, 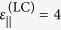 33, and the thickness of the gold nanorods/LC hybrid layer is *d* = 240 nm. The gold nanorods/LC hybrid can be regarded as a uniaxial medium with optical axis along the unit vector **n** and the geometry of its inclusion is much less than the response wavelength. Thus, the permittivity parallel to the unit vector **n** and perpendicular to the unit vector **n** of the gold nanorods/LC hybrid can be derived from the Maxwell Garnett formula (Eq. 6)^34^:


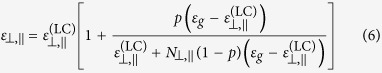


where *p* is the volume fraction of gold nanorods in LC, 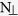 and 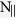 are the depolarization factors of the gold nanorods in the two directions, respectively. 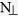 and 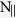 also satisfy a simple geometric rule, 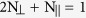, and 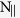
can be estimated by the following Eq. (7):


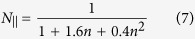


where 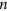 is the aspect ratio of the nanorods. Because the anisotropy of the background LC medium, we decorate the aspect ratio 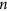 by multiplying a coefficient, 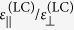. The calculated 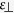 and 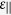 as a function of incident frequency are shown in Fig. 2(a,b). It is seen that 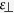 is nearly equal to 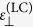 due to the small volume fraction of gold nanorods in LC, while 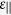 changes gradually from negative to positive and Re(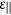) = 0 at 215 THz.

According to Eq. (2), we can obtain the permittivity in the Cartesian coordinate system (x, y, z) when the optical axis of LC tilts. As the optical axis tilts different angles, the gold nanorods/LC hybrid possesses different permittivity distributions, which makes the effective impedance of the absorber change with the tilt angle. To illustrate this, we calculate the effective impedance at 215 THz as a function of tilt angle 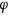 in Fig. 2(c). It is seen that the effective impedance of the absorber matches to the free space value when the tilt angle *φ* = 20°, at which a total absorption will occur. When the tilt angle takes change, however, the effective impedance match can’t be achieved. Thus, the absorber presents different absorption characters at different tilt angles. Figure 2(d) shows the simulation result of the absorption intensity at different tilt angles for different incident frequencies by using the calculated permittivity according to Eq. (2). It is seen that the abruption absorption peak gradually appears at around 215 THz as the tilt angle increases. At the tilt angle 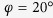, the absorber shows a total absorption effect. As the tilt angle further increases, however, the intensity of absorption peak begins to decline. The intensity change of absorption peak can be attributed to the change of effective impedance, which is consistent with the curve in Fig. 2(c).

**Figure 3:**
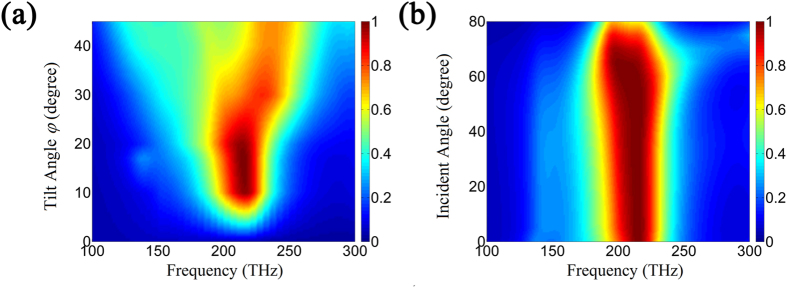
(**a**) The 3D simulated absorption intensity of the gold nanorods/LC hybrid absorber (*p* = 0.021) at different tilt angle *φ* and incident frequency; (**b**) The 3D simulated absorption intensity of the gold nanorods/LC hybrid absorber (*p* = 0.021) at different incident angle and incident frequency.

Besides the simulation based on the effective medium approximation, we also carried a 3D simulation about the absorption behavior of the gold nanorods/LC hybrid absorber. In the simulation, the nanorods are arranged in hexagonal structure and the space between adjacent nanorods is 60 nm. Figure 3(a) shows the 3D simulated absorption intensity of the gold nanorods/LC hybrid absorber at different tilt angle *φ* and incident frequency. It can be seen that the simulation results are consistent with the calculation results based on the effective medium approximation in Fig. 2(d). The total absorption is achieved when the tilt angle 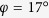, which slightly departs from 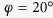. This departure may be attributed to the weak nonlocal effect of electric field distribution^35^. Furthermore, the intensity of absorption peak varies with the change of tilt angle 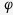, which is also consistent with the calculation results based on the effective medium approximation in Fig. 2(d). In particular, it is very interesting that the total absorption effect of the absorber is nearly omnidirectional. As shown in Fig. 3(b), for different incident angles at 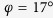, it can be seen that the absorber still keeps a total absorption effect even if the incident angle is close to 80°.

**Figure 4:**
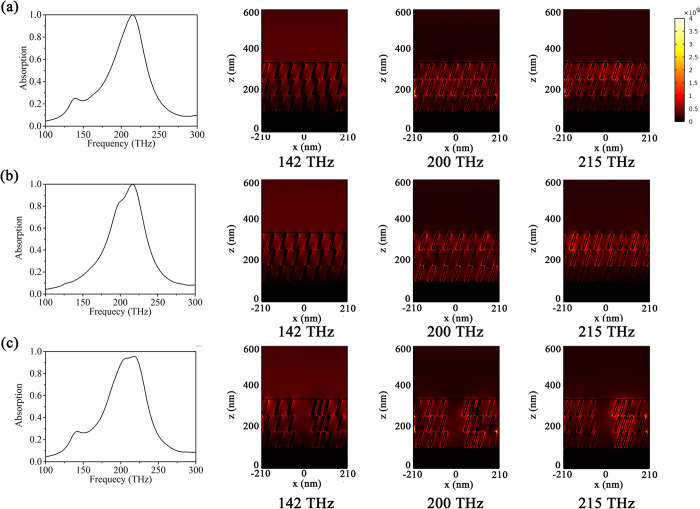
The absorption spectrum (left) of the gold nanorods/LC hybrid absorber (*p* = 0.021) at tilt angle *φ* = 17° and corresponding electric field intensity distribution (right) at 142 THz, 200 THz and 215 THz: (**a**) Nanorods are uniformly distributed; (**b**) There is misplaced arrangement of nanorods with end to end form; (**c**) There is small size of aggregation of gold nanorods.

In the above 3D simulation, the distribution of nanorods is assumed to be uniform in the model. However, in the realistic situation, the misplaced arrangement or aggregation of gold nanorods in LC is inevitable. In Fig. 4, we compare the absorption spectrum of the gold nanorods/LC hybrid absorber in the case of uniform distribution with the case of the misplaced arrangement and aggregation of gold nanorods. As shown in Fig. 4(a), in the case of uniform distribution of gold nanorods, the total absorption peak appears at 215 THz due to the strong longitudinal dipole resonance of gold nanorods (see the right electric field density distribution in Fig. 4(a)). When there is misplaced arrangement of nanorods with end to end form as shown in Fig. 4(b), it is seen that the total absorption still occurs but there is a shoulder-like absorption at 200 THz. The appearance of the shoulder-like absorption may be resulted from the electric resonance excited in the gap between nanorods due to the accumulation of polarized charges at the end of nanorods according to the field intensity distribution (see the right electric field density distribution at 200 THz in Fig. 4(b)). Compared to the electric field distribution at 215 THz, however, this resonance is not strong enough to achieve a total absorption and, as a result, it only induces the additional shoulder-like absorption. It is also worth to point out that there is a small absorption peak at 142 THz in the case of uniform distribution of nanorods in Fig. 4(a). From the electric field distribution at 142 THz, we can see that the strong field intensity appears at the diagonal end of nanorods. Thus, we assume this small absorption peak should be attributed to the dipole resonance along the diagonal line of gold nanorods when the electric field direction of incident TM wave has an angle with the gold nanorods, which further results from the cylinder end-cap^36^,^37^. The appearance of the small absorption peak is found to depend on the concentration, shape, and tilt angle of gold nanorods. In the case of misplaced arrangement of nanorods as shown in Fig. 4(b), the additional absorption peak disappears. This may be attributed to the coupling of two end-to-end nanorods^38^, leading to the change of frequency and tilt angle where the dipole resonance along the diagonal line of gold nanorods appears. Figure 4(c) shows the absorption spectrum and corresponding electric field distributions of the gold nanorods/LC hybrid absorber when there is the small size of aggregation of gold nanorods in LC. It is seen that the strong absorption is remained due to the strong electric dipole resonance at 200 THz and 215 THz and the absorption intensity slightly decreases. This indicates that the small local aggregation of gold nanorods will not significantly influence the optical property and the absorption effect has a tolerance to the deviation from uniform distribution. This can be attributed to the fact that the effective medium approximation is still valid when the aggregation size of nanorods is much smaller than wavelength. As the size of aggregation becomes larger and exceeds the incident wavelength, however, the effective medium approximation will be invalid and, as a result, the absorption intensity drops (not shown here). Therefore, to maintain a good total absorption behavior, it is necessary to avoid large size of aggregation of nanorods in LC during fabrication. One effective way to avoid large nanorod aggregations is to increase the colloid repulsive force of nanorods through surface charged or to increase the compatibility of nanorods with LC by decorating LC molecule onto the surface of gold nanorods. In addition, the small additional absorption peak at 142 THz still appears in the gold nanorods/LC hybrid absorber with small size of nanorod aggregation (see Fig. 4(c)). This can be the fact that the concentration, shape, and tilt angle of gold nanorods have not changed in the case of small size of aggregation of nanorods.

**Figure 5:**
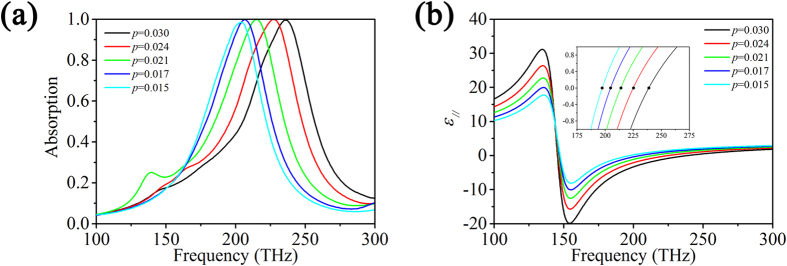
(**a**) The absorption spectrum of the gold nanorods/LC hybrid absorber containing different volume fraction of nanorods at tilt angle *φ* = 17°; (**b**) The real part of *ε*_||_ of the gold nanorods/LC hybrid containing different volume fraction of nanorods.

The concentration or volume fraction of nanorods also has an influence on the total absorption behavior as shown in Fig. 5(a). It is found that the position of total absorption peak shifts towards higher frequency with the increase of the volume fraction of gold nanorods at tilt angle 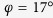. This can be attributed to the fact that the permittivity of the gold nanorods/LC hybrid has a dependence on the volume fraction of gold nanorods according to Eq. (6). Figure 5(b) presents the calculated permittivity of the gold nanorods/LC hybrid as a function of the volume fraction of gold nanorods. It can be seen that the position of 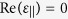 for achieving the total absorption has a red shift with the decrease of volume fraction and, as a result, the total absorption peak shifts towards higher frequency. In addition, the small additional absorption peak at low frequency due to the dipole resonance along the diagonal line of gold nanorods is obvious only in the case of volume fraction of nanorods *p* = 0.021. This can be the fact that the small additional absorption peak depends on the concentration, shape and tilt angle of gold nanorods. The tilt angle for the appearance of the small additional absorption peak is different for different volume fraction of gold nanorods/LC hybrid.

**Figure 6:**
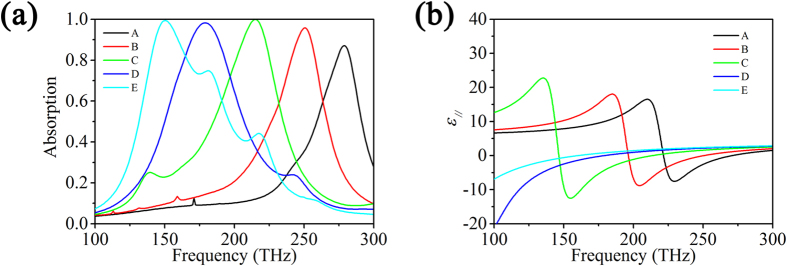
(**a**) The absorption spectrum of the gold nanorods/LC hybrid absorber (*p* = 0.021, *r* = 4.95 nm) as a function of the length of gold nanorods at *φ* = 17°; (**b**) The real part of *ε*_||_ of gold nanorods/LC hybrid (*p* = 0.021, *r* = 4.95 nm) as a function of the length of gold nanorods. (Line A–E presents *l* = 46.2 nm, 57.5 nm, 77.0 nm, 115 nm and 231 nm, respectively).

Finally, the aspect ratio of gold nanorods has an influence on the absorption. Figure 6(a) shows the absorption spectrum of the gold nanorods/LC hybrid absorber as a function of the length of gold nanorods at 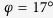, while the other conditions are fixed. It is seen that the total absorption peak shifts towards lower frequency as the length or the aspect ratio increases. This is due to the fact that the depolarization factor of gold nanorods is different at different aspect ratios, which leads to the change of permittivity of hybrid and, as a result, the impedance of the absorber varies with the aspect ratio of gold nanorods. As shown in Fig. 6(b), the real part of 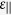 shifts to the lower frequency with the increase of length or aspect ratio of nanorods and thus the frequency for impedance match shifts to lower frequency. Therefore, the total absorption peak shifts towards lower frequency.

The fact that the total absorption depends on the aspect ratio of gold nanorods gives us a chance to realize a broadband absorption through using different length of nanorods in LC. We simulated the typical absorption when five lengths of gold nanorods (*l* = 46.2 nm, 57.5 nm, 77.0 nm, 115 nm and 231 nm) are used. In such hybrid, the effect permittivity along the optical axis can be calculated by the following Eq. (8).


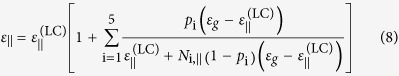


**Figure 7:**
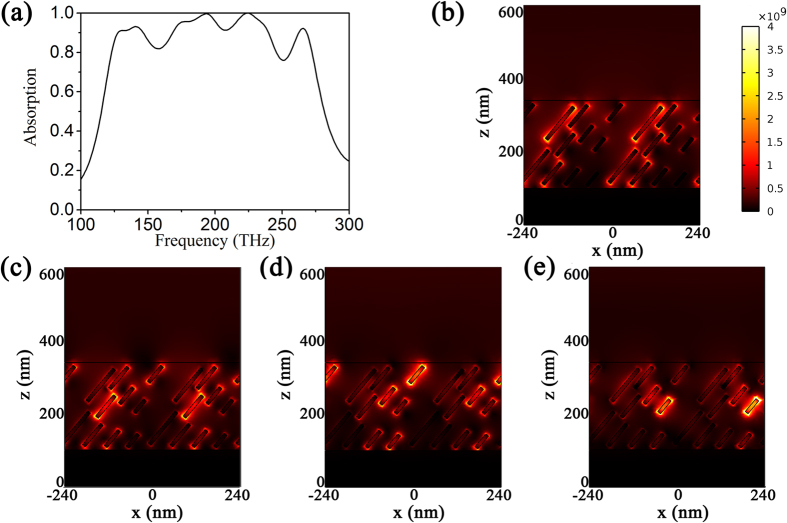
(**a**) The broadband total absorption of the gold nanorods/LC hybrid absorber containing different length of gold nanorods; (**b**–**e**) The distribution of electric field intensity at four typical absorption positions: 141 THz (**b**), 193 THz (**c**), 224 THz (**d**), and 266 THz (**e**).

Thus, at multiple frequencies, the effective permittivity of the hybrid can lead to a total absorption. Figure 7(a) shows the absorption spectrum of the absorber containing different length of nanorods at tilt angle 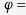 40°. The volume fraction of gold nanorods with *l* = 46.2 nm, 57.5 nm, 77 nm, 115 nm, and 231 nm is *p* = 0.005, 0.005, 0.004, and 0.002, respectively. As we expect, a broadband total absorption appears. The absorption bandwidth with absorptivity >80% is close to 150 THz. To further clarify the mechanism behind the broadband absorption, we surveyed the distribution of electric field intensity at four typical absorption positions in Fig. 7(b–e). It can be seen that the different absorption position is induced by the electric dipole resonance of different length of nanorods. Therefore, the broadband absorption is achieved due to electric resonance induced by different nanorods.

## Discussion

In this article, we have designed a soft metamterial absorber based on gold nanorods/LC hybrid. By applying an external electric field, the optical axis of the hybrid medium can be adjusted because the alignment of the nanorods agrees with the direction of LC molecule. Under different tilt angles of LC, the gold nanorods/LC hybrid exhibits different permittivity and, as a result, the absorber shows different absorption character. The effective impedance of the hybrid has been calculated as function of tilt angle 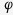. It demonstrates that the effective impedance of the absorber can match with the free-space impedance at a proper tilt angle 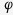 and, as a result, a total absorption occurs. Meanwhile, 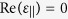 at the frequency of appearance of total absorption peak. Therefore, the hybrid medium can be also regarded as an asymmetric 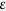
–near-zero medium at this frequency^39^,^40^,^41^. The 3D simulation results show that the total absorption of the typical gold nanorods/LC hybrid absorber (*p* = 0.021) can be realized at 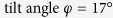, which is close to the simulation value (*φ* = 20°) based on the effective medium approximation. Furthermore, the absorber has a high absorption performance even if the incident angle exceeds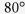, indicating the total absorption is nearly omnidirectional. In addition, the position of absorption peak has a dependence on the volume fraction and aspect ratio of gold nanorods but it has a tolerance to the deviation from uniform distribution. By using different length of gold nanorods, we have realized a useful broadband total absorption. Thus, the flexible, tunable, omnidirectional, and broadband characteristics make the soft gold nanorods/LC hybrid absorber be a good candidate for the development of smart sensor, energy harvest or energy conversion devices.

## Methods

Based on finite element method, we use COMSOL Multiphysics 4.4 software to simulate the absorption property of the absorber. In our simulation, the relative permittivity of gold nanorods (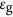) is obtained from Drude mode, 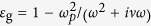 with plasma frequency 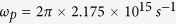 and collision frequency 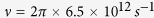 42,43. The relative permittivity of LC is set as changing with tilt angle of LC 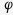:


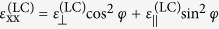



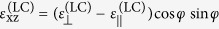



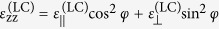


Because electrons experience additional scattering resulting from the metal surfaces^44^,^45^, we use a collision frequency value that is three times as large as that in bulk. The incident wave is applied by a port boundary. And the magnetic field component of the incident wave is along y axis, setting as 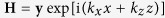. Periodic boundary conditions are also used along x axis. Reflection R is calculated from the software. Because the thickness of the gold film is enough to eliminate the transmission, the absorption A is derived from *A* = 1 − *R*. In the 3D simulation, we simplify the gold nanorod as a cylinder with the same radius and length.

## Additional Information

**How to cite this article**: Su, Z. *et al.* Soft and broadband infrared metamaterial absorber based on gold nanorod/liquid crystal hybrid with tunable total absorption. *Sci. Rep.* **5**, 16698; doi: 10.1038/srep16698 (2015).
